# Bis[*N*-(2-hy­droxy­eth­yl)-*N*-methyl­dithio­carbamato-κ*S*][2,4,6-tris­(pyridin-2-yl)-1,3,5-triazine-κ^3^ *N* ^1^,*N* ^2^,*N* ^6^]zinc dioxane sesquisolvate

**DOI:** 10.1107/S160053681200671X

**Published:** 2012-02-24

**Authors:** Hadi D. Arman, Pavel Poplaukhin, Edward R. T. Tiekink

**Affiliations:** a Department of Chemistry, The University of Texas at San Antonio, One UTSA Circle, San Antonio, Texas 78249-0698, USA; b Chemical Abstracts Service, 2540 Olentangy River Road, Columbus, Ohio 43202, USA; c Department of Chemistry, University of Malaya, 50603 Kuala Lumpur, Malaysia

## Abstract

The asymmetric unit of the title compound, [Zn(C_4_H_8_NOS_2_)_2_(C_18_H_12_N_6_)]·1.5C_4_H_8_O_2_, comprises a Zn-containing mol­ecule and one and a half dioxane mol­ecules as one of the solvent mol­ecules is located about a crystallographic inversion centre. The approximately square-pyramidal N_3_S_2_ donor set is defined by two monodentate dithio­carbamate ligands and two pyridine and one triazine N atom from the tridentate triazine ligand. Mol­ecules are connected into a supra­molecular array *via* O—H⋯S and O—H⋯N hydrogen bonds. These stack along the *b* axis and the solvent mol­ecules reside in the channels thus formed.

## Related literature

For background on structural studies on hydroxyl-substituted dithio­carbamate ligands, see: Benson *et al.* (2007 ▶); Poplaukhin & Tiekink (2010 ▶). For the coordination modes of triazine mol­ecules, see: Therrin (2011 ▶). For additional structural analysis, see: Addison *et al.* (1984 ▶); Spek (2009 ▶).
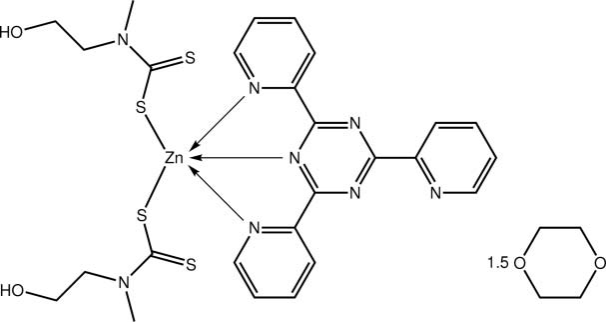


## Experimental

### 

#### Crystal data

[Zn(C_4_H_8_NOS_2_)_2_(C_18_H_12_N_6_)]·1.5C_4_H_8_O_2_
*M* *_r_* = 810.33
Triclinic, 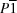

*a* = 11.863 (10) Å
*b* = 13.019 (11) Å
*c* = 13.199 (11) Å
α = 107.214 (12)°
β = 105.780 (15)°
γ = 100.892 (11)°
*V* = 1792 (3) Å^3^
*Z* = 2
Mo *K*α radiation
μ = 0.97 mm^−1^
*T* = 98 K
0.40 × 0.30 × 0.05 mm

#### Data collection

Rigaku AFC12/SATURN724 diffractometer
Absorption correction: multi-scan (*ABSCOR*; Higashi, 1995 ▶) *T*_min_ = 0.539, *T*_max_ = 1
11537 measured reflections
6940 independent reflections
5803 reflections with *I* > 2σ(*I*)
*R*_int_ = 0.067

#### Refinement

*R*[*F* ^2^ > 2σ(*F* ^2^)] = 0.085
*wR*(*F* ^2^) = 0.222
*S* = 1.12
6940 reflections
459 parameters
2 restraints
H atoms treated by a mixture of independent and constrained refinement
Δρ_max_ = 1.54 e Å^−3^
Δρ_min_ = −1.08 e Å^−3^

### 

Data collection: *CrystalClear* (Molecular Structure Corporation & Rigaku, 2005 ▶); cell refinement: *CrystalClear*; data reduction: *CrystalClear*; program(s) used to solve structure: *SHELXS97* (Sheldrick, 2008 ▶); program(s) used to refine structure: *SHELXL97* (Sheldrick, 2008 ▶); molecular graphics: *ORTEP-3* (Farrugia, 1997 ▶) and *DIAMOND* (Brandenburg, 2006 ▶); software used to prepare material for publication: *publCIF* (Westrip, 2010 ▶).

## Supplementary Material

Crystal structure: contains datablock(s) global, I. DOI: 10.1107/S160053681200671X/lh5418sup1.cif

Structure factors: contains datablock(s) I. DOI: 10.1107/S160053681200671X/lh5418Isup2.hkl

Additional supplementary materials:  crystallographic information; 3D view; checkCIF report

## Figures and Tables

**Table 1:** Selected bond lengths (Å)

Zn—S1	2.335 (2)
Zn—S3	2.368 (2)
Zn—N3	2.082 (5)
Zn—N6	2.211 (5)
Zn—N7	2.249 (5)

**Table 2:** Hydrogen-bond geometry (Å, °)

*D*—H⋯*A*	*D*—H	H⋯*A*	*D*⋯*A*	*D*—H⋯*A*
O1—H1O⋯N4^i^	0.84 (7)	2.34 (8)	3.038 (8)	141 (7)
O1—H1O⋯N8^i^	0.84 (7)	2.27 (6)	2.995 (8)	146 (7)
O2—H2O⋯S2^ii^	0.84 (9)	2.65 (8)	3.387 (6)	149 (8)

Symmetry codes: (i) 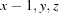; (ii) 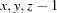.

**Fig. 1.**
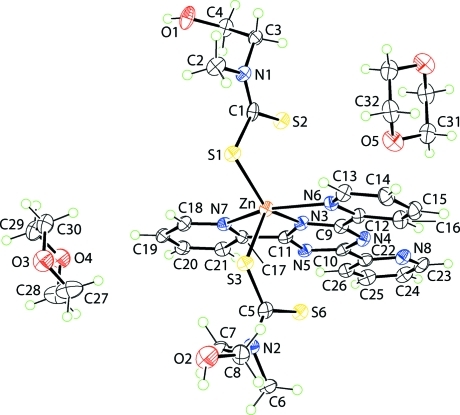
Molecular structure of (I) showing atom-labelling scheme and displacement ellipsoids at the 50% probability level. The O5-dioxane molecule is centrosymmetric and the unlabelled atoms are related by 1 - x, y, 1 - z.

**Fig. 2.**
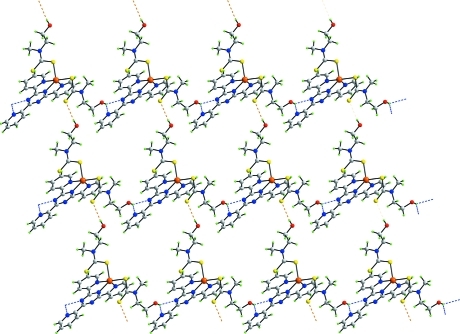
Supramolecular layer in the ac plane in (I). The arrays are mediated by O–H···S and O—H···N hydrogen bonds which are shown as orange and blue dashed lines, respectively.

**Fig. 3.**
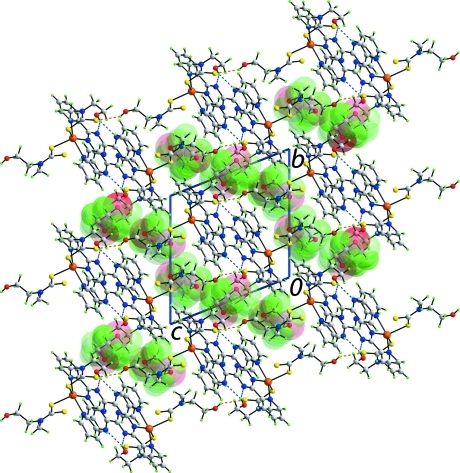
Unit-cell contents in (I) viewed in projection down the a axis. The dioxane molecules are presented in space filling mode.

## supplementary crystallographic information

### Comment

The title compound, (I), was prepared in the context of previous crystal engineering studies of zinc dithiocarbamates (Benson *et al.*, 2007; Poplaukhin & Tiekink, 2010).

The asymmetric unit of (I) comprises a Zn-containing molecule and one and half dioxane molecules as one dioxane molecule is located about a centre on inversion. The Zn^II^ atom, Fig. 1, is coordinated by two monodentate dithiocarbamate ligands and three N donors from the 2,4,6-tris(pyridin-2-yl)-1,3,5-triazine molecule. The monodentate mode of coordination of the dithiocarbamate ligands is confirmed by the disparity in the C—S bond lengths, Table 1, with the shorter lengths associated with the formally thione C═S bonds. The Zn···S2 and Zn···S4 separations are 3.210 (3) and 3.590 (3) Å, respectively. The observed tridentate mode of coordination of the triazine molecule is often observed in its metal complexes (Therrin, 2011).

The resultant N_3_S_2_ donor set defines a square pyramid. This assignment is based on the value calculated for τ of 0.07 for the Zn atom, which compares to the τ values of 0.0 and 1.0 for ideal square pyramidal and trigonal bipyramidal geometries, respectively (Spek, 2009; Addison *et al.*, 1984).

The presence of O—H···S and O—H···N hydrogen bonding leads to supramolecular layers in the *ac* plane, Fig. 2 and Table 2. The dioxane molecules occupy channels in the crystal structure as highlighted in Fig. 3.

### Experimental

The title compound was prepared by dissolving zinc(II) bis[*N*-(2-hydroxyethyl)-*N*-methyldithiocarbamato-κ*S*]zinc(II) (Benson *et al.*, 2007; 0.5 mmol, 184 mg) and 2,4,6-tris(pyridin-2-yl)-1,3,5-triazine (0.5 mmol, 160 mg) into a methanol/ethanol solution. The solution made an abrupt colour change from yellow to red. Suitable X-ray quality crystals were grown by liquid diffusion of dioxane into the methanol/ethanol solution.

### Refinement

C-bound H-atoms were placed in calculated positions (C—H 0.95–0.99 Å) and were included in the refinement in the riding model approximation with *U*_iso_(H) set to 1.2–1.5*U*_eq_(C). The O-bound H-atoms were located in a difference Fourier map and refined with an O—H restraint of 0.84±0.01 Å, and with *U*_iso_(H) = 1.5*U*_eq_(O). The maximum and minimum residual electron density peaks of 1.54 and 1.08 e Å^-3^, respectively, were located 0.92 Å and 0.96 Å from the Zn atom.

### Crystal data

**Table:**

[Zn(C_4_H_8_NOS_2_)_2_(C_18_H_12_N_6_)]·1.5C_4_H_8_O_2_	*Z* = 2
*M**_r_* = 810.33	*F*(000) = 844
Triclinic, *P*1	*D*_x_ = 1.502 Mg m^−^^3^
Hall symbol: -P 1	Mo *K*α radiation, λ = 0.71069 Å
*a* = 11.863 (10) Å	Cell parameters from 7249 reflections
*b* = 13.019 (11) Å	θ = 2.1–40.5°
*c* = 13.199 (11) Å	µ = 0.97 mm^−^^1^
α = 107.214 (12)°	*T* = 98 K
β = 105.780 (15)°	Prism, red
γ = 100.892 (11)°	0.40 × 0.30 × 0.05 mm
*V* = 1792 (3) Å^3^	

### Data collection

**Table:**

Rigaku AFC12/SATURN724 diffractometer	6940 independent reflections
Radiation source: fine-focus sealed tube	5803 reflections with *I* > 2σ(*I*)
Graphite monochromator	*R*_int_ = 0.067
ω scans	θ_max_ = 26.0°, θ_min_ = 2.1°
Absorption correction: multi-scan (*ABSCOR*; Higashi, 1995)	*h* = −13→14
*T*_min_ = 0.539, *T*_max_ = 1	*k* = −16→15
11537 measured reflections	*l* = −16→16

### Refinement

**Table:**

Refinement on *F*^2^	Primary atom site location: structure-invariant direct methods
Least-squares matrix: full	Secondary atom site location: difference Fourier map
*R*[*F*^2^ > 2σ(*F*^2^)] = 0.085	Hydrogen site location: inferred from neighbouring sites
*wR*(*F*^2^) = 0.222	H atoms treated by a mixture of independent and constrained refinement
*S* = 1.12	*w* = 1/[σ^2^(*F*_o_^2^) + (0.1081*P*)^2^ + 2.961*P*] where *P* = (*F*_o_^2^ + 2*F*_c_^2^)/3
6940 reflections	(Δ/σ)_max_ < 0.001
459 parameters	Δρ_max_ = 1.54 e Å^−^^3^
2 restraints	Δρ_min_ = −1.08 e Å^−^^3^

### Special details

**Table:**

Geometry. All s.u.'s (except the s.u. in the dihedral angle between two l.s. planes) are estimated using the full covariance matrix. The cell s.u.'s are taken into account individually in the estimation of s.u.'s in distances, angles and torsion angles; correlations between s.u.'s in cell parameters are only used when they are defined by crystal symmetry. An approximate (isotropic) treatment of cell s.u.'s is used for estimating s.u.'s involving l.s. planes.
Refinement. Refinement of *F*^2^ against ALL reflections. The weighted *R*-factor *wR* and goodness of fit *S* are based on *F*^2^, conventional *R*-factors *R* are based on *F*, with *F* set to zero for negative *F*^2^. The threshold expression of *F*^2^ > 2σ(*F*^2^) is used only for calculating *R*-factors(gt) *etc*. and is not relevant to the choice of reflections for refinement. *R*-factors based on *F*^2^ are statistically about twice as large as those based on *F*, and *R*- factors based on ALL data will be even larger.

### Fractional atomic coordinates and isotropic or equivalent isotropic displacement parameters (Å^2^)

**Table:**

	*x*	*y*	*z*	*U*_iso_*/*U*_eq_	
Zn	0.32652 (5)	0.25219 (5)	0.17902 (5)	0.0214 (2)	
S1	0.11993 (12)	0.16780 (12)	0.14083 (11)	0.0249 (3)	
S2	0.25672 (12)	0.18343 (12)	0.37341 (12)	0.0258 (3)	
S3	0.30899 (11)	0.26302 (11)	−0.00035 (11)	0.0235 (3)	
S4	0.58474 (12)	0.35416 (12)	0.10628 (11)	0.0255 (3)	
O1	−0.1375 (4)	0.1443 (4)	0.3793 (4)	0.0365 (10)	
H1O	−0.154 (7)	0.203 (4)	0.409 (6)	0.055*	
O2	0.2186 (4)	0.2066 (5)	−0.3764 (4)	0.0436 (11)	
H2O	0.243 (8)	0.229 (7)	−0.422 (6)	0.065*	
N1	0.0198 (4)	0.0710 (4)	0.2576 (4)	0.0227 (9)	
N2	0.4548 (4)	0.3517 (4)	−0.0923 (4)	0.0235 (9)	
N3	0.4988 (4)	0.3147 (4)	0.3053 (4)	0.0217 (9)	
N4	0.6911 (4)	0.2907 (4)	0.3819 (4)	0.0232 (9)	
N5	0.6605 (4)	0.4724 (4)	0.4388 (4)	0.0223 (9)	
N6	0.4064 (4)	0.1104 (4)	0.1621 (4)	0.0222 (9)	
N7	0.3506 (4)	0.4350 (4)	0.2704 (4)	0.0206 (9)	
N8	0.9181 (4)	0.3772 (4)	0.5458 (4)	0.0273 (10)	
C1	0.1241 (5)	0.1345 (4)	0.2602 (4)	0.0227 (11)	
C2	−0.0933 (5)	0.0332 (5)	0.1600 (5)	0.0298 (12)	
H2A	−0.0774	−0.0020	0.0907	0.045*	
H2B	−0.1225	0.0978	0.1555	0.045*	
H2C	−0.1557	−0.0216	0.1683	0.045*	
C3	0.0098 (5)	0.0464 (4)	0.3564 (5)	0.0243 (11)	
H3A	0.0884	0.0376	0.3975	0.029*	
H3B	−0.0544	−0.0260	0.3305	0.029*	
C4	−0.0215 (5)	0.1375 (5)	0.4374 (5)	0.0286 (12)	
H4A	−0.0237	0.1186	0.5043	0.034*	
H4B	0.0414	0.2107	0.4636	0.034*	
C5	0.4525 (5)	0.3262 (4)	−0.0006 (5)	0.0242 (11)	
C6	0.5716 (5)	0.4002 (5)	−0.1017 (5)	0.0278 (12)	
H6A	0.6225	0.4632	−0.0311	0.042*	
H6B	0.5564	0.4271	−0.1646	0.042*	
H6C	0.6141	0.3427	−0.1155	0.042*	
C7	0.3460 (5)	0.3288 (5)	−0.1898 (5)	0.0259 (11)	
H7A	0.3554	0.3924	−0.2164	0.031*	
H7B	0.2737	0.3241	−0.1658	0.031*	
C8	0.3233 (5)	0.2221 (5)	−0.2863 (5)	0.0319 (13)	
H8A	0.3949	0.2260	−0.3114	0.038*	
H8B	0.3119	0.1576	−0.2612	0.038*	
C9	0.5757 (5)	0.2521 (4)	0.3114 (4)	0.0214 (10)	
C10	0.7283 (5)	0.4012 (4)	0.4461 (4)	0.0226 (11)	
C11	0.5469 (5)	0.4246 (4)	0.3676 (4)	0.0203 (10)	
C12	0.5231 (5)	0.1356 (4)	0.2277 (4)	0.0207 (10)	
C13	0.3513 (5)	0.0074 (4)	0.0824 (4)	0.0240 (11)	
H13	0.2675	−0.0110	0.0376	0.029*	
C14	0.4121 (5)	−0.0733 (5)	0.0630 (5)	0.0306 (12)	
H14	0.3713	−0.1455	0.0052	0.037*	
C15	0.5332 (5)	−0.0466 (5)	0.1295 (5)	0.0299 (12)	
H15	0.5773	−0.1001	0.1168	0.036*	
C16	0.5909 (5)	0.0594 (5)	0.2156 (5)	0.0266 (11)	
H16	0.6734	0.0787	0.2641	0.032*	
C17	0.4612 (5)	0.4928 (4)	0.3488 (4)	0.0208 (10)	
C18	0.2691 (5)	0.4911 (5)	0.2475 (5)	0.0244 (11)	
H18	0.1908	0.4512	0.1907	0.029*	
C19	0.2962 (5)	0.6065 (5)	0.3048 (5)	0.0263 (11)	
H19	0.2370	0.6444	0.2871	0.032*	
C20	0.4107 (5)	0.6656 (5)	0.3879 (5)	0.0274 (12)	
H20	0.4301	0.7439	0.4293	0.033*	
C21	0.4961 (5)	0.6073 (5)	0.4091 (5)	0.0257 (11)	
H21	0.5761	0.6452	0.4635	0.031*	
C22	0.8523 (5)	0.4487 (5)	0.5334 (5)	0.0252 (11)	
C23	1.0287 (5)	0.4190 (5)	0.6270 (5)	0.0311 (13)	
H23	1.0770	0.3695	0.6352	0.037*	
C24	1.0766 (5)	0.5305 (5)	0.6997 (5)	0.0313 (13)	
H24	1.1547	0.5561	0.7577	0.038*	
C25	1.0089 (5)	0.6035 (6)	0.6864 (6)	0.0361 (14)	
H25	1.0403	0.6807	0.7344	0.043*	
C26	0.8950 (5)	0.5636 (5)	0.6028 (5)	0.0277 (12)	
H26	0.8464	0.6124	0.5921	0.033*	
O3	−0.0197 (4)	0.6269 (4)	−0.0009 (4)	0.0421 (11)	
O4	0.1276 (4)	0.7852 (4)	0.2186 (4)	0.0417 (11)	
C27	0.1087 (7)	0.6711 (7)	0.0298 (7)	0.055 (2)	
H27A	0.1282	0.6750	−0.0375	0.066*	
H27B	0.1504	0.6206	0.0578	0.066*	
C28	0.1554 (8)	0.7883 (7)	0.1218 (7)	0.058 (2)	
H28A	0.2452	0.8175	0.1427	0.070*	
H28B	0.1170	0.8398	0.0925	0.070*	
C29	−0.0024 (6)	0.7421 (6)	0.1860 (6)	0.0411 (15)	
H29A	−0.0424	0.7930	0.1571	0.049*	
H29B	−0.0235	0.7391	0.2528	0.049*	
C30	−0.0481 (6)	0.6276 (6)	0.0968 (6)	0.0377 (14)	
H30A	−0.0111	0.5762	0.1275	0.045*	
H30B	−0.1381	0.5994	0.0755	0.045*	
O5	0.5355 (5)	0.0717 (4)	0.4454 (4)	0.0447 (11)	
C31	0.6229 (6)	0.0446 (6)	0.5217 (6)	0.0406 (15)	
H31A	0.6998	0.1075	0.5588	0.049*	
H31B	0.6414	−0.0231	0.4798	0.049*	
C32	0.4249 (6)	−0.0228 (6)	0.3903 (6)	0.0392 (15)	
H32A	0.4432	−0.0904	0.3480	0.047*	
H32B	0.3624	−0.0058	0.3360	0.047*	

### Atomic displacement parameters (Å^2^)

**Table:**

	*U*^11^	*U*^22^	*U*^33^	*U*^12^	*U*^13^	*U*^23^
Zn	0.0220 (3)	0.0205 (3)	0.0169 (3)	0.0061 (2)	0.0055 (2)	0.0016 (2)
S1	0.0243 (6)	0.0279 (7)	0.0193 (7)	0.0064 (5)	0.0074 (5)	0.0052 (5)
S2	0.0231 (6)	0.0283 (7)	0.0184 (7)	0.0056 (5)	0.0034 (5)	0.0026 (5)
S3	0.0229 (6)	0.0249 (6)	0.0196 (7)	0.0050 (5)	0.0074 (5)	0.0048 (5)
S4	0.0253 (6)	0.0282 (7)	0.0194 (7)	0.0069 (5)	0.0069 (5)	0.0051 (6)
O1	0.031 (2)	0.042 (2)	0.027 (2)	0.0203 (19)	0.0060 (18)	−0.0025 (19)
O2	0.036 (2)	0.065 (3)	0.025 (2)	0.015 (2)	0.0059 (19)	0.013 (2)
N1	0.023 (2)	0.022 (2)	0.019 (2)	0.0065 (17)	0.0078 (18)	0.0016 (18)
N2	0.028 (2)	0.026 (2)	0.015 (2)	0.0092 (19)	0.0071 (18)	0.0063 (19)
N3	0.024 (2)	0.018 (2)	0.020 (2)	0.0056 (17)	0.0103 (18)	0.0004 (18)
N4	0.026 (2)	0.025 (2)	0.017 (2)	0.0108 (18)	0.0096 (18)	0.0023 (19)
N5	0.023 (2)	0.021 (2)	0.022 (2)	0.0050 (18)	0.0103 (18)	0.0042 (18)
N6	0.028 (2)	0.025 (2)	0.015 (2)	0.0104 (18)	0.0086 (18)	0.0084 (18)
N7	0.024 (2)	0.021 (2)	0.017 (2)	0.0063 (17)	0.0095 (17)	0.0036 (18)
N8	0.027 (2)	0.034 (3)	0.018 (2)	0.010 (2)	0.0082 (19)	0.005 (2)
C1	0.026 (2)	0.020 (2)	0.019 (3)	0.014 (2)	0.007 (2)	−0.002 (2)
C2	0.026 (3)	0.035 (3)	0.019 (3)	0.005 (2)	0.005 (2)	0.001 (2)
C3	0.027 (3)	0.025 (3)	0.024 (3)	0.011 (2)	0.013 (2)	0.008 (2)
C4	0.029 (3)	0.032 (3)	0.020 (3)	0.011 (2)	0.006 (2)	0.004 (2)
C5	0.031 (3)	0.019 (2)	0.020 (3)	0.010 (2)	0.011 (2)	0.000 (2)
C6	0.023 (3)	0.036 (3)	0.025 (3)	0.007 (2)	0.011 (2)	0.011 (3)
C7	0.026 (3)	0.034 (3)	0.020 (3)	0.010 (2)	0.010 (2)	0.011 (2)
C8	0.037 (3)	0.037 (3)	0.022 (3)	0.016 (3)	0.009 (2)	0.009 (3)
C9	0.024 (2)	0.024 (3)	0.012 (2)	0.007 (2)	0.006 (2)	0.002 (2)
C10	0.023 (2)	0.025 (3)	0.017 (3)	0.005 (2)	0.011 (2)	0.001 (2)
C11	0.024 (2)	0.019 (2)	0.016 (3)	0.005 (2)	0.010 (2)	0.001 (2)
C12	0.026 (2)	0.021 (2)	0.013 (2)	0.010 (2)	0.008 (2)	0.002 (2)
C13	0.025 (2)	0.023 (3)	0.017 (3)	0.006 (2)	0.005 (2)	0.000 (2)
C14	0.041 (3)	0.024 (3)	0.027 (3)	0.013 (2)	0.013 (3)	0.007 (2)
C15	0.039 (3)	0.026 (3)	0.027 (3)	0.016 (2)	0.015 (3)	0.006 (2)
C16	0.032 (3)	0.024 (3)	0.020 (3)	0.010 (2)	0.006 (2)	0.002 (2)
C17	0.027 (3)	0.016 (2)	0.020 (3)	0.009 (2)	0.012 (2)	0.005 (2)
C18	0.027 (3)	0.024 (3)	0.022 (3)	0.010 (2)	0.012 (2)	0.004 (2)
C19	0.029 (3)	0.028 (3)	0.028 (3)	0.014 (2)	0.017 (2)	0.010 (2)
C20	0.034 (3)	0.025 (3)	0.026 (3)	0.010 (2)	0.016 (2)	0.008 (2)
C21	0.025 (3)	0.024 (3)	0.022 (3)	0.003 (2)	0.007 (2)	0.004 (2)
C22	0.025 (3)	0.029 (3)	0.022 (3)	0.007 (2)	0.010 (2)	0.009 (2)
C23	0.028 (3)	0.042 (3)	0.026 (3)	0.015 (3)	0.014 (2)	0.010 (3)
C24	0.020 (2)	0.045 (3)	0.017 (3)	0.004 (2)	0.001 (2)	0.004 (3)
C25	0.034 (3)	0.036 (3)	0.031 (3)	0.004 (3)	0.011 (3)	0.006 (3)
C26	0.029 (3)	0.029 (3)	0.020 (3)	0.006 (2)	0.009 (2)	0.005 (2)
O3	0.036 (2)	0.049 (3)	0.027 (2)	0.007 (2)	0.0007 (19)	0.007 (2)
O4	0.041 (2)	0.047 (3)	0.032 (3)	0.011 (2)	0.009 (2)	0.012 (2)
C27	0.050 (4)	0.069 (5)	0.041 (4)	0.001 (4)	0.022 (4)	0.016 (4)
C28	0.056 (5)	0.064 (5)	0.042 (4)	−0.005 (4)	0.017 (4)	0.017 (4)
C29	0.038 (3)	0.041 (4)	0.047 (4)	0.019 (3)	0.018 (3)	0.013 (3)
C30	0.036 (3)	0.038 (3)	0.043 (4)	0.017 (3)	0.015 (3)	0.013 (3)
O5	0.059 (3)	0.037 (2)	0.032 (3)	0.011 (2)	0.012 (2)	0.009 (2)
C31	0.037 (3)	0.038 (3)	0.042 (4)	0.014 (3)	0.010 (3)	0.010 (3)
C32	0.045 (4)	0.035 (3)	0.026 (3)	0.016 (3)	0.002 (3)	0.004 (3)

### Geometric parameters (Å, º)

**Table:**

Zn—S1	2.335 (2)	C10—C22	1.478 (7)
Zn—S3	2.368 (2)	C11—C17	1.490 (7)
Zn—N3	2.082 (5)	C12—C16	1.390 (7)
Zn—N6	2.211 (5)	C13—C14	1.385 (8)
Zn—N7	2.249 (5)	C13—H13	0.9500
S1—C1	1.744 (6)	C14—C15	1.378 (8)
S2—C1	1.701 (5)	C14—H14	0.9500
S3—C5	1.746 (6)	C15—C16	1.399 (8)
S4—C5	1.692 (6)	C15—H15	0.9500
O1—C4	1.415 (7)	C16—H16	0.9500
O1—H1O	0.840 (10)	C17—C21	1.382 (7)
O2—C8	1.399 (7)	C18—C19	1.394 (8)
O2—H2O	0.840 (10)	C18—H18	0.9500
N1—C1	1.338 (7)	C19—C20	1.391 (8)
N1—C2	1.461 (7)	C19—H19	0.9500
N1—C3	1.461 (7)	C20—C21	1.394 (8)
N2—C5	1.352 (7)	C20—H20	0.9500
N2—C7	1.461 (7)	C21—H21	0.9500
N2—C6	1.464 (7)	C22—C26	1.407 (8)
N3—C11	1.337 (7)	C23—C24	1.386 (9)
N3—C9	1.335 (7)	C23—H23	0.9500
N4—C9	1.327 (7)	C24—C25	1.374 (9)
N4—C10	1.348 (7)	C24—H24	0.9500
N5—C11	1.317 (7)	C25—C26	1.378 (8)
N5—C10	1.345 (7)	C25—H25	0.9500
N6—C12	1.335 (7)	C26—H26	0.9500
N6—C13	1.339 (7)	O3—C27	1.417 (8)
N7—C17	1.336 (7)	O3—C30	1.417 (8)
N7—C18	1.341 (7)	O4—C28	1.413 (9)
N8—C23	1.336 (7)	O4—C29	1.431 (8)
N8—C22	1.342 (7)	C27—C28	1.525 (11)
C2—H2A	0.9800	C27—H27A	0.9900
C2—H2B	0.9800	C27—H27B	0.9900
C2—H2C	0.9800	C28—H28A	0.9900
C3—C4	1.523 (7)	C28—H28B	0.9900
C3—H3A	0.9900	C29—C30	1.487 (9)
C3—H3B	0.9900	C29—H29A	0.9900
C4—H4A	0.9900	C29—H29B	0.9900
C4—H4B	0.9900	C30—H30A	0.9900
C6—H6A	0.9800	C30—H30B	0.9900
C6—H6B	0.9800	O5—C31	1.413 (8)
C6—H6C	0.9800	O5—C32	1.451 (8)
C7—C8	1.500 (8)	C31—C32^i^	1.498 (10)
C7—H7A	0.9900	C31—H31A	0.9900
C7—H7B	0.9900	C31—H31B	0.9900
C8—H8A	0.9900	C32—C31^i^	1.498 (10)
C8—H8B	0.9900	C32—H32A	0.9900
C9—C12	1.479 (7)	C32—H32B	0.9900
			
N3—Zn—N6	73.74 (17)	N6—C12—C9	114.7 (4)
N3—Zn—N7	73.82 (16)	C16—C12—C9	122.7 (5)
N6—Zn—N7	147.55 (17)	N6—C13—C14	122.3 (5)
N3—Zn—S1	143.15 (13)	N6—C13—H13	118.8
N6—Zn—S1	103.52 (13)	C14—C13—H13	118.8
N7—Zn—S1	102.90 (12)	C15—C14—C13	118.5 (5)
N3—Zn—S3	118.69 (13)	C15—C14—H14	120.7
N6—Zn—S3	97.83 (12)	C13—C14—H14	120.7
N7—Zn—S3	96.73 (12)	C14—C15—C16	119.8 (5)
S1—Zn—S3	98.16 (5)	C14—C15—H15	120.1
C1—S1—Zn	102.41 (18)	C16—C15—H15	120.1
C5—S3—Zn	110.22 (19)	C12—C16—C15	117.6 (5)
C4—O1—H1O	116 (6)	C12—C16—H16	121.2
C8—O2—H2O	106 (6)	C15—C16—H16	121.2
C1—N1—C2	121.4 (5)	N7—C17—C21	123.8 (5)
C1—N1—C3	121.7 (4)	N7—C17—C11	114.9 (4)
C2—N1—C3	116.5 (4)	C21—C17—C11	121.3 (5)
C5—N2—C7	124.2 (4)	N7—C18—C19	121.7 (5)
C5—N2—C6	120.4 (4)	N7—C18—H18	119.1
C7—N2—C6	115.3 (4)	C19—C18—H18	119.1
C11—N3—C9	116.3 (4)	C20—C19—C18	119.5 (5)
C11—N3—Zn	121.2 (4)	C20—C19—H19	120.2
C9—N3—Zn	121.1 (3)	C18—C19—H19	120.2
C9—N4—C10	114.9 (4)	C19—C20—C21	118.4 (5)
C11—N5—C10	114.6 (4)	C19—C20—H20	120.8
C12—N6—C13	119.1 (5)	C21—C20—H20	120.8
C12—N6—Zn	116.0 (3)	C17—C21—C20	118.2 (5)
C13—N6—Zn	124.4 (4)	C17—C21—H21	120.9
C17—N7—C18	118.3 (4)	C20—C21—H21	120.9
C17—N7—Zn	115.2 (3)	N8—C22—C26	122.5 (5)
C18—N7—Zn	126.3 (4)	N8—C22—C10	117.2 (5)
C23—N8—C22	117.5 (5)	C26—C22—C10	120.3 (5)
N1—C1—S2	123.0 (4)	N8—C23—C24	123.5 (6)
N1—C1—S1	117.0 (4)	N8—C23—H23	118.2
S2—C1—S1	119.9 (3)	C24—C23—H23	118.2
N1—C2—H2A	109.5	C25—C24—C23	118.7 (5)
N1—C2—H2B	109.5	C25—C24—H24	120.6
H2A—C2—H2B	109.5	C23—C24—H24	120.6
N1—C2—H2C	109.5	C24—C25—C26	119.3 (6)
H2A—C2—H2C	109.5	C24—C25—H25	120.4
H2B—C2—H2C	109.5	C26—C25—H25	120.4
N1—C3—C4	113.1 (4)	C25—C26—C22	118.4 (6)
N1—C3—H3A	109.0	C25—C26—H26	120.8
C4—C3—H3A	109.0	C22—C26—H26	120.8
N1—C3—H3B	109.0	C27—O3—C30	109.4 (5)
C4—C3—H3B	109.0	C28—O4—C29	108.4 (5)
H3A—C3—H3B	107.8	O3—C27—C28	110.7 (7)
O1—C4—C3	108.2 (4)	O3—C27—H27A	109.5
O1—C4—H4A	110.1	C28—C27—H27A	109.5
C3—C4—H4A	110.1	O3—C27—H27B	109.5
O1—C4—H4B	110.1	C28—C27—H27B	109.5
C3—C4—H4B	110.1	H27A—C27—H27B	108.1
H4A—C4—H4B	108.4	O4—C28—C27	110.6 (6)
N2—C5—S4	120.1 (4)	O4—C28—H28A	109.5
N2—C5—S3	116.9 (4)	C27—C28—H28A	109.5
S4—C5—S3	123.0 (3)	O4—C28—H28B	109.5
N2—C6—H6A	109.5	C27—C28—H28B	109.5
N2—C6—H6B	109.5	H28A—C28—H28B	108.1
H6A—C6—H6B	109.5	O4—C29—C30	110.5 (5)
N2—C6—H6C	109.5	O4—C29—H29A	109.5
H6A—C6—H6C	109.5	C30—C29—H29A	109.5
H6B—C6—H6C	109.5	O4—C29—H29B	109.5
N2—C7—C8	113.2 (5)	C30—C29—H29B	109.5
N2—C7—H7A	108.9	H29A—C29—H29B	108.1
C8—C7—H7A	108.9	O3—C30—C29	112.0 (6)
N2—C7—H7B	108.9	O3—C30—H30A	109.2
C8—C7—H7B	108.9	C29—C30—H30A	109.2
H7A—C7—H7B	107.7	O3—C30—H30B	109.2
O2—C8—C7	109.8 (5)	C29—C30—H30B	109.2
O2—C8—H8A	109.7	H30A—C30—H30B	107.9
C7—C8—H8A	109.7	C31—O5—C32	108.4 (5)
O2—C8—H8B	109.7	O5—C31—C32^i^	110.3 (5)
C7—C8—H8B	109.7	O5—C31—H31A	109.6
H8A—C8—H8B	108.2	C32^i^—C31—H31A	109.6
N4—C9—N3	123.9 (5)	O5—C31—H31B	109.6
N4—C9—C12	122.0 (5)	C32^i^—C31—H31B	109.6
N3—C9—C12	113.9 (4)	H31A—C31—H31B	108.1
N5—C10—N4	125.1 (5)	O5—C32—C31^i^	109.1 (5)
N5—C10—C22	116.6 (5)	O5—C32—H32A	109.9
N4—C10—C22	118.2 (5)	C31^i^—C32—H32A	109.9
N5—C11—N3	124.8 (5)	O5—C32—H32B	109.9
N5—C11—C17	120.9 (4)	C31^i^—C32—H32B	109.9
N3—C11—C17	114.3 (4)	H32A—C32—H32B	108.3
N6—C12—C16	122.6 (5)		
			
N3—Zn—S1—C1	−10.2 (3)	C9—N4—C10—N5	−3.5 (8)
N6—Zn—S1—C1	70.6 (2)	C9—N4—C10—C22	175.0 (4)
N7—Zn—S1—C1	−90.3 (2)	C10—N5—C11—N3	−0.2 (7)
S3—Zn—S1—C1	170.79 (17)	C10—N5—C11—C17	−179.0 (4)
N3—Zn—S3—C5	−0.1 (2)	C9—N3—C11—N5	−4.9 (8)
N6—Zn—S3—C5	−75.8 (2)	Zn—N3—C11—N5	−171.2 (4)
N7—Zn—S3—C5	75.1 (2)	C9—N3—C11—C17	173.9 (4)
S1—Zn—S3—C5	179.26 (19)	Zn—N3—C11—C17	7.6 (6)
N6—Zn—N3—C11	171.7 (4)	C13—N6—C12—C16	0.9 (8)
N7—Zn—N3—C11	−7.3 (4)	Zn—N6—C12—C16	−170.8 (4)
S1—Zn—N3—C11	−97.3 (4)	C13—N6—C12—C9	178.9 (5)
S3—Zn—N3—C11	81.6 (4)	Zn—N6—C12—C9	7.1 (6)
N6—Zn—N3—C9	6.1 (4)	N4—C9—C12—N6	−179.2 (5)
N7—Zn—N3—C9	−172.9 (4)	N3—C9—C12—N6	−2.2 (7)
S1—Zn—N3—C9	97.1 (4)	N4—C9—C12—C16	−1.2 (8)
S3—Zn—N3—C9	−84.0 (4)	N3—C9—C12—C16	175.8 (5)
N3—Zn—N6—C12	−7.1 (4)	C12—N6—C13—C14	−2.0 (8)
N7—Zn—N6—C12	−5.3 (5)	Zn—N6—C13—C14	169.0 (4)
S1—Zn—N6—C12	−149.0 (3)	N6—C13—C14—C15	0.9 (9)
S3—Zn—N6—C12	110.6 (4)	C13—C14—C15—C16	1.2 (9)
N3—Zn—N6—C13	−178.4 (4)	N6—C12—C16—C15	1.2 (8)
N7—Zn—N6—C13	−176.6 (4)	C9—C12—C16—C15	−176.7 (5)
S1—Zn—N6—C13	39.7 (4)	C14—C15—C16—C12	−2.2 (8)
S3—Zn—N6—C13	−60.7 (4)	C18—N7—C17—C21	0.9 (8)
N3—Zn—N7—C17	5.7 (3)	Zn—N7—C17—C21	176.0 (4)
N6—Zn—N7—C17	3.9 (5)	C18—N7—C17—C11	−178.9 (4)
S1—Zn—N7—C17	147.7 (3)	Zn—N7—C17—C11	−3.8 (5)
S3—Zn—N7—C17	−112.3 (3)	N5—C11—C17—N7	176.9 (5)
N3—Zn—N7—C18	−179.7 (4)	N3—C11—C17—N7	−2.0 (7)
N6—Zn—N7—C18	178.5 (4)	N5—C11—C17—C21	−2.9 (8)
S1—Zn—N7—C18	−37.6 (4)	N3—C11—C17—C21	178.2 (5)
S3—Zn—N7—C18	62.4 (4)	C17—N7—C18—C19	−1.4 (8)
C2—N1—C1—S2	178.2 (4)	Zn—N7—C18—C19	−175.8 (4)
C3—N1—C1—S2	5.0 (7)	N7—C18—C19—C20	0.1 (8)
C2—N1—C1—S1	−1.2 (6)	C18—C19—C20—C21	1.7 (8)
C3—N1—C1—S1	−174.4 (4)	N7—C17—C21—C20	0.8 (8)
Zn—S1—C1—N1	−171.5 (3)	C11—C17—C21—C20	−179.4 (5)
Zn—S1—C1—S2	9.0 (3)	C19—C20—C21—C17	−2.1 (8)
C1—N1—C3—C4	85.4 (6)	C23—N8—C22—C26	−0.6 (8)
C2—N1—C3—C4	−88.1 (6)	C23—N8—C22—C10	−178.0 (5)
N1—C3—C4—O1	62.1 (6)	N5—C10—C22—N8	176.3 (5)
C7—N2—C5—S4	178.7 (4)	N4—C10—C22—N8	−2.4 (7)
C6—N2—C5—S4	2.1 (7)	N5—C10—C22—C26	−1.1 (7)
C7—N2—C5—S3	−0.7 (7)	N4—C10—C22—C26	−179.8 (5)
C6—N2—C5—S3	−177.3 (4)	C22—N8—C23—C24	1.6 (8)
Zn—S3—C5—N2	−171.4 (3)	N8—C23—C24—C25	−1.8 (9)
Zn—S3—C5—S4	9.2 (4)	C23—C24—C25—C26	1.1 (9)
C5—N2—C7—C8	−97.2 (6)	C24—C25—C26—C22	−0.2 (9)
C6—N2—C7—C8	79.5 (6)	N8—C22—C26—C25	0.0 (8)
N2—C7—C8—O2	−179.5 (5)	C10—C22—C26—C25	177.2 (5)
C10—N4—C9—N3	−2.3 (7)	C30—O3—C27—C28	−55.7 (8)
C10—N4—C9—C12	174.4 (5)	C29—O4—C28—C27	−58.8 (8)
C11—N3—C9—N4	6.3 (8)	O3—C27—C28—O4	58.6 (9)
Zn—N3—C9—N4	172.5 (4)	C28—O4—C29—C30	59.0 (8)
C11—N3—C9—C12	−170.7 (4)	C27—O3—C30—C29	56.7 (7)
Zn—N3—C9—C12	−4.4 (6)	O4—C29—C30—O3	−58.9 (7)
C11—N5—C10—N4	4.7 (7)	C32—O5—C31—C32^i^	−60.6 (7)
C11—N5—C10—C22	−173.9 (4)	C31—O5—C32—C31^i^	59.8 (7)

Symmetry code: (i) −*x*+1, −*y*, −*z*+1.

### Hydrogen-bond geometry (Å, º)

**Table:**

*D*—H···*A*	*D*—H	H···*A*	*D*···*A*	*D*—H···*A*
O1—H1*O*···N4^ii^	0.84 (7)	2.34 (8)	3.038 (8)	141 (7)
O1—H1*O*···N8^ii^	0.84 (7)	2.27 (6)	2.995 (8)	146 (7)
O2—H2*O*···S2^iii^	0.84 (9)	2.65 (8)	3.387 (6)	149 (8)

Symmetry codes: (ii) *x*−1, *y*, *z*; (iii) *x*, *y*, *z*−1.
